# LPMO *Af*AA9_B and Cellobiohydrolase *Af*Cel6A from *A. fumigatus* Boost Enzymatic Saccharification Activity of Cellulase Cocktail

**DOI:** 10.3390/ijms22010276

**Published:** 2020-12-29

**Authors:** Aline Vianna Bernardi, Luis Eduardo Gerolamo, Paula Fagundes de Gouvêa, Deborah Kimie Yonamine, Lucas Matheus Soares Pereira, Arthur Henrique Cavalcante de Oliveira, Sérgio Akira Uyemura, Taisa Magnani Dinamarco

**Affiliations:** 1 Faculdade de Filosofia Ciências e Letras de Ribeirão Preto, Universidade de São Paulo, Ribeirão Preto 14040-901, Brazil; alinevbernardi@gmail.com (A.V.B.); gerolamo00@hotmail.com (L.E.G.); paulafgouvea@yahoo.com.br (P.F.d.G.); deborah.yonamine@hotmail.com (D.K.Y.); lucas.matheus.pereira@usp.br (L.M.S.P.); arthurdeoliveira@ffclrp.usp.br (A.H.C.d.O.); 2 Faculdade de Ciências Farmacêuticas de Ribeirão Preto, Universidade de São Paulo, Ribeirão Preto 14040-903, Brazil; suyemura@fcfrp.usp.br

**Keywords:** GH6 cellobiohydrolase, AA9 LPMO, lignocellulose hydrolysis, bioethanol

## Abstract

Cellulose is the most abundant polysaccharide in lignocellulosic biomass, where it is interlinked with lignin and hemicellulose. Bioethanol can be produced from biomass. Since breaking down biomass is difficult, cellulose-active enzymes secreted by filamentous fungi play an important role in degrading recalcitrant lignocellulosic biomass. We characterized a cellobiohydrolase (*Af*Cel6A) and lytic polysaccharide monooxygenase LPMO (*Af*AA9_B) from *Aspergillus fumigatus* after they were expressed in *Pichia pastoris* and purified. The biochemical parameters suggested that the enzymes were stable; the optimal temperature was ~60 °C. Further characterization revealed high turnover numbers (*k_cat_* of 147.9 s^−1^ and 0.64 s^−1^, respectively). Surprisingly, when combined, *Af*Cel6A and *Af*AA9_B did not act synergistically. *Af*Cel6A and *Af*AA9_B association inhibited *Af*Cel6A activity, an outcome that needs to be further investigated. However, *Af*Cel6A or *Af*AA9_B addition boosted the enzymatic saccharification activity of a cellulase cocktail and the activity of cellulase *Af*-EGL7. Enzymatic cocktail supplementation with *Af*Cel6A or *Af*AA9_B boosted the yield of fermentable sugars from complex substrates, especially sugarcane exploded bagasse, by up to 95%. The synergism between the cellulase cocktail and *Af*AA9_B was enzyme- and substrate-specific, which suggests a specific enzymatic cocktail for each biomass by up to 95%. The synergism between the cellulase cocktail and *Af*AA9_B was enzyme- and substrate-specific, which suggests a specific enzymatic cocktail for each biomass.

## 1. Introduction

Fossil fuel depletion, increasing energy consumption, growing CO_2_ emissions, and climate change have increased the demand for renewable energy sources. In this scenario, lignocellulosic residues stand out as a new generation of renewable energy sources, including second-generation (2G) ethanol [1,2,3,4,5]. Lignocellulosic biomass-derived biofuels can potentially substitute fossil fuels with the advantage that they can help to reduce the emission of greenhouse gases and global warming [6,7]. Every year, tons of agricultural residues, such as byproducts of sugarcane, corn, wheat, rice, and barley, are generated worldwide and have emerged as the most promising feedstock to produce biofuels by hydrolysis and subsequent fermentation [8].

The composition of the plant cell wall varies in terms of the percentage of cellulose (35–50%), hemicellulose (20–30%), and lignin (20–30%). The wall lignocellulosic structure is recalcitrant and resists chemical and biological treatments. Cellulose, a crystalline homopolysaccharide, is made up of thousands of D-glucose subunits linked by β-1,4-glycosidic bonds, forming linear chains. The cellulose chains are bound through intra- and intermolecular hydrogen bonds, creating insoluble microfibrils [9]. The recalcitrant structure of the plant cell wall matrix makes the release of soluble sugars challenging [10].

Industrial processes that produce ethanol from cellulose require that mixtures of fungal cellulases be employed, so that soluble sugars are released for further fermentation into bioethanol [7,11]. These enzymes work synergistically to break down polysaccharides and crystalline cellulose [12,13]. First, endoglucanases (EGL, EC 3.2.1.4) hydrolyze β-1,4-glucosidic bonds in amorphous regions of the cellulose chains, to release cello-oligosaccharides; cellobiohydrolases (CBH; EC 3.2.1.91) act on short cellulose molecules and cello-oligosaccharides, releasing disaccharide units like cellobiose. Then, β-glucosidases (BG; EC 3.2.1.21) cleave cellobiose into glucose for further fermentation. Together, these enzymes are part of an enzymatic cocktail and are used to break down lignocellulose.

In contrast to cellulases, lytic polysaccharide monooxygenases (LPMO; EC: 1.14.99.53–56) degrade cellulose by an oxidative mechanism and enhance accessibility to cellulose, improving the hydrolytic performance of cellulases [14,15,16]. LPMOs are copper-dependent enzymes that act on crystalline cellulose and other polysaccharides in nature, to generate oxidized and non-oxidized chain ends. In addition, LPMO is a virulence factor in fungal meningitis [17].

The fact that LPMO boosts the activity of hydrolytic enzymes during chitin degradation was first described in 2005 [18]. The LPMO activity on cellulose and other biomasses has also been reported [19,20]. The copper ion in the LPMO catalytic structure is coordinated to three nitrogen atoms of the two conserved histidine residues in a histidine brace, which is essential for LPMO activity [21,22,23,24,25,26,27]. The LPMO oxidative mechanism is not fully understood, but analysis of reaction products has revealed that LPMO hydroxylates carbon C1 or C4, or both. To initiate oxidative cleavage, an enzyme, such as cellobiose dehydrogenase, or a small reductor molecule must reduce the LPMO copper center. Subsequently, the enzyme reacts with a co-substrate (O_2_ or H_2_O_2_), to form oxygen species that can hydroxylate C1 or C4 in the glycosidic bond [28,29].

Some studies have described inhibitory results or no synergism between LPMOs and cellulases. For example, *Hj*LPMO9A addition to accellerase elicits no synergism until 100 h [30]. Moreover, *Nc*LPMO9F reduces CBHI efficiency in the degradation of mixed amorphous-crystalline cellulosic substrate (MACS) [31]. *Mt*LPMO9L affects CBHI and CBHII differently depending on the ratio between the enzymes, substrate characteristics, and incubation time. These data highlight that understanding the synergistic mechanism between LPMO and GHs is still necessary and will be helpful for the development of novel cellulase mixtures.

Enzymes from thermophilic microorganisms offer several advantages for industrial applications. For example, *Aspergillus fumigatus* produces thermophilic CAZymes, which have high cellulolytic activity and stability in a wide range of pH and at elevated temperatures, unlike commercial fungal cellulases [32,33,34,35].

To characterize the association of cellulases (*Af*Cel6A and *Af*-EGL7) and LPMO (*Af*AA9_B) from *A. fumigatus*, we evaluated their action on the degradation of different biomasses on a pilot scale. *Af*Cel6A is a cellobiohydrolase from the glycoside hydrolase (GH) class, family 6; it acts exclusively on nonreducing ends of cellulosic polymers. *Af*-EGL7 is a previously characterized endoglucanase that can potentially hydrolyze biomass [32,36].

Here, we present the biochemical characterization of *Af*Cel6A and *Af*AA9_B after they are expressed in *Pichia pastoris.* We will show that supplementation of enzymatic cocktails can enhance the production of fermentable sugars, and that LPMOs have a critical role in biomass hydrolysis. In addition, we evaluate the synergistic effect between *Af*AA9_B and cellulases (*Af*Cel6A and *Af*-EGL7) and show different effects for the two enzymes.

## 2. Results and Discussion

Enzymatic biomass hydrolysis underlies most of the cost involved in biofuel production [37,38]. Different commercially available cellulolytic cocktails such as Novozyme, Du-Pont-Genencor, and Dyadic are still expensive. These cocktails consist of several enzymes that promote complete lignocellulosic biomass conversion into fermentable sugars [39,40]. However, widely variable biomasses are available for biorefinery purposes; e.g., wheat straw, rice straw, corncob, cotton-stalk, and sugar cane bagasse, so these commercial cocktails may not have the same efficiency for all feedstocks [41].

Developing cheaper and more effective enzymatic cocktails for hydrolysis of different biomasses is one of the major interests of researchers devoted to biomass conversion. Such cocktails can only be achieved by reducing the amount of enzymes that is required for hydrolysis, by bioprospecting and characterizing new enzymes, and by developing new enzyme mixtures. [42]. Moreover, the addition of an extra enzyme increases hydrolysis performance by increasing the release of fermentable sugars and reducing the time of hydrolysis.

LPMO (*Af*AA9_B) and Cellobiohydrolase GH6 (*Af*Cel6A) from *A. fumigatus* and expressed in *Escherichia coli* and *Aspergillus oryzae*, respectively, have been described [35,43]. However, to evaluate the action of the combined enzymes, we characterized and analyzed their biochemical properties after expressing them in *Pichia pastoris*, and we detected some differences.

### 2.1. Expression and Purification of Recombinant AfCel6A and AfAA9_B

**Figure 1:**
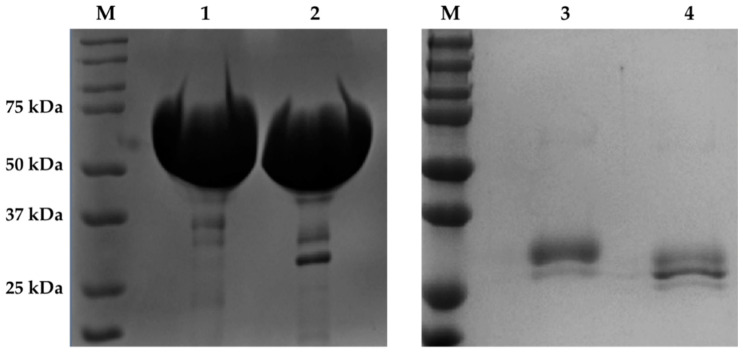
SDS-PAGE (10% polyacrylamide gel) analysis of the purified recombinant *Af*Cel6A and *Af*AA9_B. Lane M, molecular mass standards (Precision™ Protein Standards Dual Color—BioRad); lane 1, purified recombinant *Af*Cel6A; lane 2, deglycosylated *Af*Cel6A; lane 3, purified recombinant *Af*AA9_B; lane 4, deglycosylated *Af*AA9_B.

We successfully expressed recombinant *Af*Cel6A and *Af*AA9_B in *P. pastoris* X-33. After induction for 144 h, we collected, concentrated, and purified the culture supernatants on Ni^+^ Sepharose 6 Fast Flow resin (Ge Healthcare, Little Chalfont, UK). SDS-PAGE revealed that the purified recombinant *Af*Cel6A and *Af*AA9_B had apparent molecular masses of approximately 65 and 30 kDa, respectively (Figure 1). After Endo H treatment, the molecular mass of *Af*Cel6A remained almost the same, while *Af*AA9_B migrated as a band of approximately 26 kDa. Analyses of potential N-glycosylation sites by the NetNGlyc 1.0 program (http://www.cbs.dtu.dk/services/NetNG lyc/) suggested that a potential site was present at position N413 in *Af*Cel6A and N159 in *Af*AA9_B, confirmed by deglycosylation of the recombinant proteins by the enzyme Endoglycosidase H. The presence of N-glycans at different sites in the structure of the enzyme can influence enzymatic properties, such as secretion, folding, and stability, among others [44].

We excised the purified *Af*AA9_B from the gel and analyzed it on the LC-MS/MS Xevo TQS (Waters) system at the Multi-User Laboratory of Mass Spectrometry, which confirmed that the enzyme was LPMO (Table 1).

### 2.2. Structural Analysis and Predictions by Circular Dichroism (CD)

**Figure 2:**
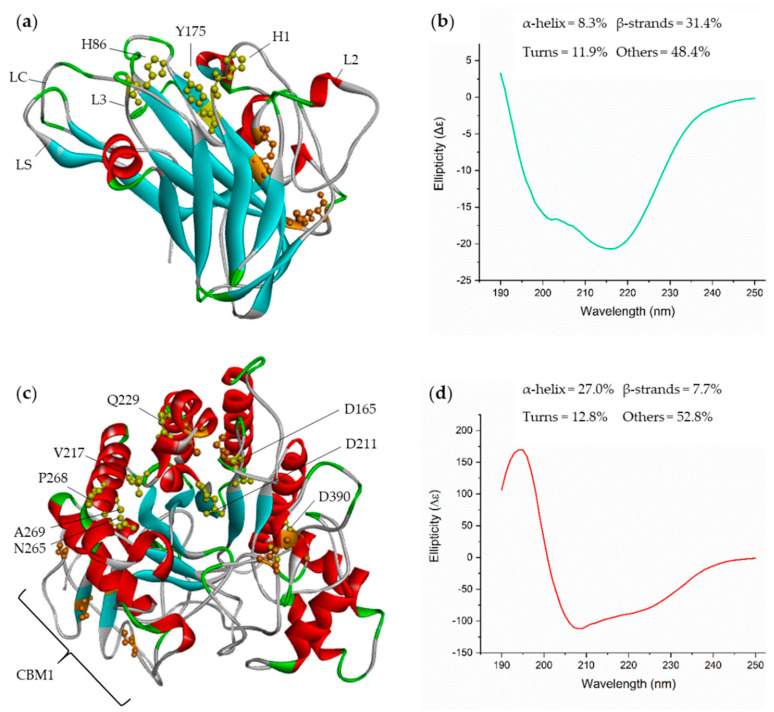
Ribbon representation of the enzymes *Af*AA9_B (**a**) and *Af*Cel6A (**c**) and their main conserved residues and structures. Active site residues are represented in yellow, and disulfide bonds are represented in orange. *Af*AA9_B loops are represented by LC (C-terminus), LS (short), L3, and L2. CBM1 residues are indicated by a brace in *Af*Cel6A. Circular dichroism spectra obtained from 190 to 250 nm (UV-distant) for *Af*AA9_B with Cu(II) (**b**) and *Af*Cel6A (**d**) at 25 °C. Both enzymes were in 20 mM sodium phosphate buffer (pH = 7.4), and the spectra were read by using a quartz cuvette with an optical path of 0.1 cm. The mean spectra for each sample were normalized by subtracting the buffer spectrum.

LPMOs comprise a group of redox enzymes that belong to the auxiliary activity (AA) class (families 9–16, except 12) [45] and which bear a β-sandwich core (presence of 8–10 β-strands). The catalytic region of the enzyme is known as histidine brace [21,24,46], which contains many loops and accounts for the active site topology and substrate specificity. Specificity is due to the presence of aromatic residues and their weak interactions with polysaccharides [22,47]. Figure 2a shows the crystallized structure of LPMO *Af*AA9_B (PDB: 5 × 6A), where the active site residues H1, H86, and Y175 in the histidine brace are highlighted.

Due to its tunnel-shaped catalytic structure, *Af*Cel6A acts exclusively on nonreducing ends of cellulosic polymers. The cellulosic polymers enter this catalytic structure through one of their extremities, and *Af*Cel6A continuously cleaves the long chains into small cellobiose units via anomeric inversion (Figure 2c). The enzymatic core consists of a distorted α/β-barrel motif. Few parallel β-strands in sandwich conformation are connected by several loops, which are rich in α-helices [48,49,50]. As depicted in Figure 2c, *Af*Cel6A contains N-terminal CBM1 (carbohydrate-binding module) as well as the main residues involved in catalysis, namely Q229, P268, V217, N265, A269 [48], D165, D211, and D390 (determined by the Pfam database [51]).

Since the 1980s, thousands of three-dimensional protein structures have been resolved and deposited in the Protein Data Bank (PDB), allowing more detailed insights into the structure and function of proteins, including protein complexes [52]. However, performing structural studies under the conditions in which proteins actually operate (i.e., generally in solution), as well as under other conditions, is crucial, and providing measures of the rates of structural changes in proteins, which are often essential to their biological function [52], is vital. Circular dichroism (CD) has become increasingly recognized as a valuable structural technique for addressing these issues [52]. In this sense, the first important information to be obtained is whether the structure of the expressed proteins in solution corresponds to crystal or modeled structures. To this end, we obtained the secondary structure content on the basis of on circular dichroism spectra, from which we predicted the secondary structures of the enzymes by using BESTSEL [53]. This analysis showed substantial structural similarity between the enzymes and their templates from PDB:5X6A resolved by Q. Shen (unpublished) (for *Af*AA9_B) [54] and Phyre2 web server [55] (for *Af*Cel6A), as displayed in Figure 2 and Supplementary Table S1.

The CD spectrum of *Af*AA9_B and its predicted secondary structures (Figure 2b) demonstrated that the enzyme consisted of 8.3% α-helices and 31.4% β-strands. These values reinforced that LPMOs present a large number of β-strands in their cores, reflected by the well-defined negative peak at 218 nm, the small peak at 190 nm, and the approximated single band profile. Small negative peaks around 208 nm also evidenced the small number of helices [56]. Compared to the expected values based on the PDB: 5X6A structure, the percentage of β-strands was exactly the same, while the percentage of α-helices was −4.3%. *Ta*LPMO9A (PDB: 2YET) [26], an LPMO from *Thermoascus aurantiacus*, has been reported to share 71% identity with *Af*AA9_B and to present similar proportions of α-helices and β-strands: 30.8% and 15.0%, respectively.

*Af*Cel6A presented 27.0% α-helix and 7.7% β-strands, as estimated by BeStSel (Figure 2d). The accentuated peak at 190 nm and the two negative peaks near 208 nm and 222 nm indicated a large number of α-helices. The absence of a negative peak at approximately 218 nm and a single band profile are typical of proteins with low content of β-strands [56]. On the basis of the proportions of α-helices and β-strands estimated by Phyre2 [55] and the Kabsch and Sander method [57] for the modeled structure (Figure 2c), the differences were −4.3% and −1.0%, and −1.0% and −2.3%, respectively. The enzyme Cel6A from *Trichoderma reesei* (PDB:1QJW), which shares 69% identity, presents a similar proportion of 35.8% α-helices and 8.7% β-strands [58]. Furthermore, a cellobiohydrolase from a different *A. fumigatus* strain that shares 99% identity with *Af*Cel6A consists of 26.0% α-helix and 15.4% β-strands, confirming that the prediction based on the CD spectrum is remarkably close.

Therefore, CD analysis of both enzymes obtained herein evidenced that their secondary structure profiles resembled the profiles described in the literature. This indicated that both enzymes were correctly folded during heterologous expression, and that their structures were maintained after they were purified.

Confirming that the structure of wild enzymes in solution corresponds to the structure obtained by crystallography or modeling allows enzymes to be efficiently improved by protein engineering. To increase the catalytic efficiency of cocktails, alterations modeled on the protein structure can be accompanied by spectroscopic studies in solution, allowing improved activity to be directly associated with conformational changes in the structure of the enzyme.

### 2.3. Enzymatic Properties of AfCel6A and AfAA9_B

We used CM-Cellulose and 2,6-DMP as substrates to determine the enzymatic properties of *Af*Cel6A and the activity of *Af*AA9_B, respectively.

**Figure 3:**
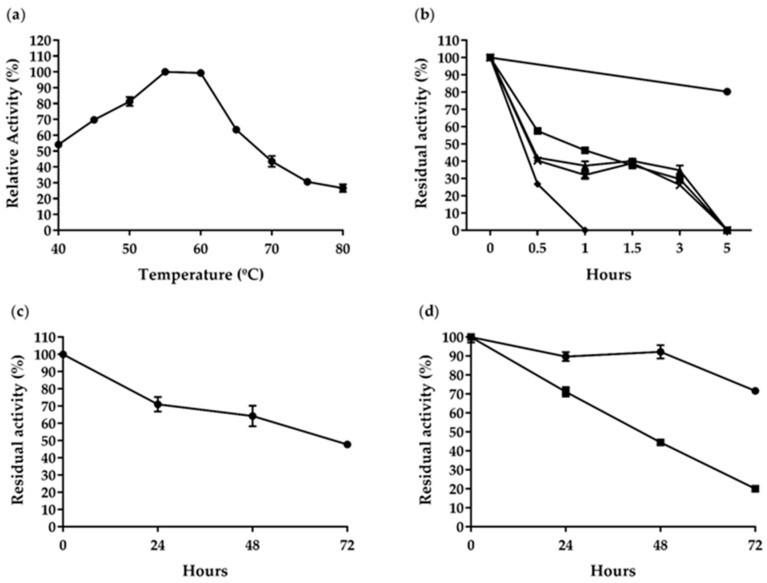
Temperature effects on *Af*Cel6A and *Af*AA9_B activity and stability. (**a**) *Af*Cel6A temperature-activity profiles at optimal pH for 45 min. (**b**) *Af*Cel6A thermostability at ● 50, ■ 60, ▲ 70, × 80, and ♦ 90 °C for different times. (**c**) *Af*Cel6A thermostability at 50 °C for up to 72 h. (**d**) *Af*AA9_B thermostability at ● 50 and ■ 60 °C for 72 h. Each value in the panel represents the mean of three experiments.

The optimal temperature for *Af*Cel6A activity was 55–60 °C, and the enzyme retained over 54% of the maximum activity between 40 and 65 °C. At 70, 75, and 80 °C, *Af*Cel6A maintained 43.5%, 30%, and 26% of the maximum activity, respectively (Figure 3a). Most characterized cellobiohydrolases, shown in Table 2, were also active at these temperatures. We studied the *Af*Cel6A thermal stability after preincubating it at 50, 60, 70, 80, or 90 °C for different times (Figure 3b). The enzyme was stable after 30 min and retained 57.5%, 42.0%, 40.4%, and 26.9% of the initial activity at 60, 70, 80, and 90 °C, respectively. *Af*Cel6A maintained about 30% of the initial activity at 60–80 °C. However, the enzyme was completely inactivated after 5 h at 60–80 °C. *Af*Cel6A was stable at 50 °C. It lost only 30% of its original activity after 24 h and retained 64.2% and 47.7% of its initial activity after 48 and 72 h, respectively (Figure 3c). These results showed that *Af*Cel6A was stable at high temperatures, especially at 50 °C. In another study, after expression in *A. oryzae*, *Af*Cel6A was stable at 60 °C, but it completely lost its activity at 70 °C [35]. Therefore, *Af*Cel6A was more stable after expression in *P. pastoris* than in *A. oryzae.*

The optimal temperature for *Af*AA9_B activity was 60 °C (data not shown). *Af*AA9_B was stable at 50 and 60 °C and retained over 75% and 20% of its initial activity, respectively (Figure 3d). Like *Af*AA9_B, other LPMOs were stable at 50 and 60 °C; e.g., PMO9D_SCYTH, PMO9D_MALCI, *Mt*LPMO9D, *Mt*LPMO9J, and *Mt*LPMO9A (Table 3).

**Figure 4:**
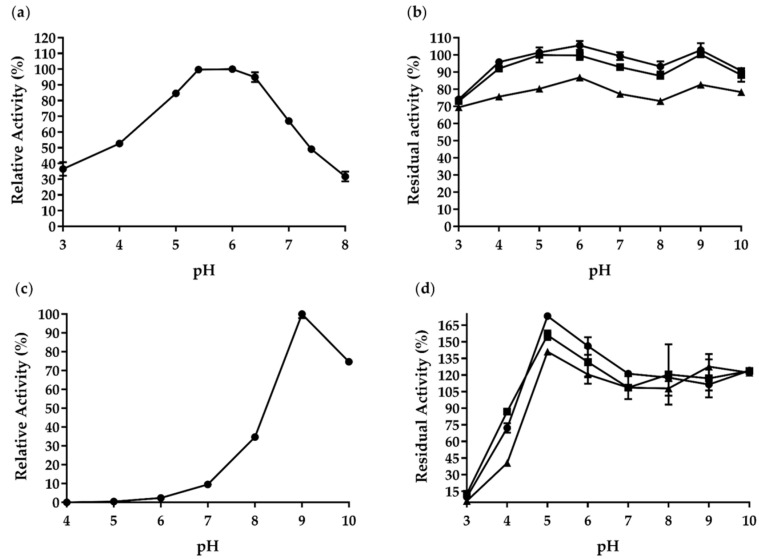
pH effects on the enzymatic activity and stability of purified recombinant *Af*Cel6A and *Af*AA9_B. (**a**) *Af*Cel6A pH-activity profile. (**b**) pH stability of *Af*Cel6A after ● 24, ■ 48, and ▲ 72 h of preincubation at 4 °C. The enzyme activities were measured under standard conditions. (**c**) *Af*AA9_B pH-activity profile. (**d**) pH stability of *Af*AA9_B after ● 24, ■ 48, and ▲ 72 h of preincubation at 4 °C. Each value in the panel represents the mean of three experiments.

Figure 4 illustrates how pH influenced *Af*Cel6A and *Af*AA9_B. The highest *Af*Cel6A activity emerged at pH 5.5–6.0, but it was active in a narrow pH range (pH 4.0–7.5) and retained >50% of maximum activity therein (Figure 4a).

Many cellobiohydrolases seem to belong to the class of acidic enzymes, with optimal pH ranging from 3.9 to 6.0; for example, CBH II from *Talaromyces emersonii* (pH 3.8), Cel6D (pH 3.9), CBH II from *Chaetomium thermophilum* (pH 4.0), CBH II from *Penicillium occitanis* (pH 3.0–5.0), CBH II from *Trichoderma viride* (pH 5.0), J11 CelA (pH 6.0), and EX4 (pH 5.0). Only one GH6 has been classified as active at pH 9.0: *Mo*Cel6A from *Magnaporthe oryzae* (Table 2).

We also investigated *Af*Cel6A pH stability (Figure 4b). Notably, *Af*Cel6A was stable at pH ranging between 3 and 10 and retained over 70% of its original activity after 72 h. Compared to other GH6 cellobiohydrolases, *Af*Cel6A was more stable over a wide pH range, whereas others had narrower range of pH stability—CBH II from *Talaromyces emersonii* (38 min at pH 5.0), Cel6D (over 60% activity at pH 4.0–6.0 and 47 °C and complete inactivation at pH 4.0 and 55 °C), CBH II from *Penicillium occitanis* (24 h at pH 2.0–9.0), J11 CelA (1 h), and EX4 (over 80% activity at pH 3.0–8.0 at 60 °C for 1 h).

*Af*AA9_B showed the highest activity at pH 9.0. At pH 10.0, it retained >74.0% of its activity (Figure 4c). The optimal *Af*AA9_B pH was pH 9.0, but this enzyme was stable at pH ranging between 5.0 and 10.0 and maintained 100% of the original activity after 72 h (Figure 4d). Compared to PMO9D_SCYTH (pH 7.0) and PMO9D_MALCI (pH 9.0), *Af*AA9_B was more stable, whereas the former LPMOs were stable at a specific pH (Table 3).

### 2.4. Substrate Specificity and Kinetic Parameters

**Figure 5:**
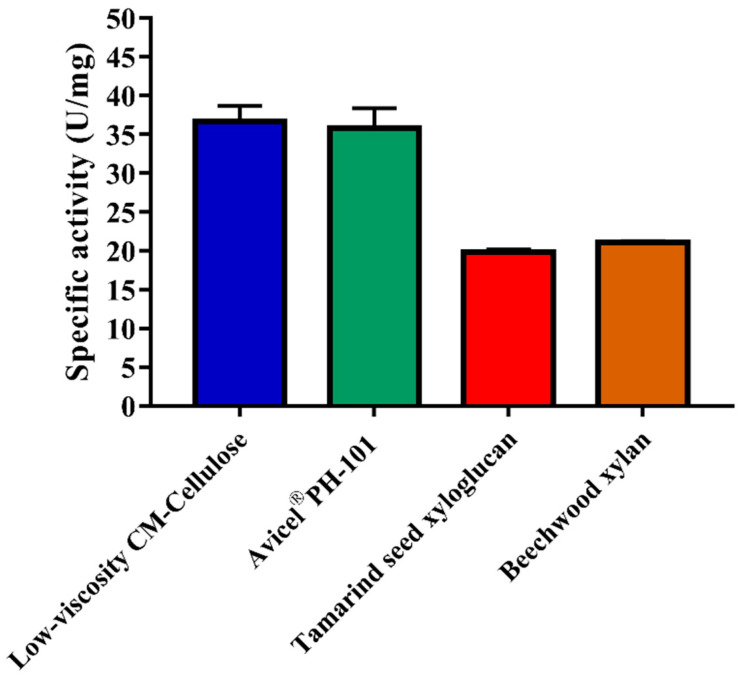
*Af*Cel6A specific activity (U mg^−1^) toward CM-Cellulose, Avicel, xyloglucan, and xylan. Each reaction was performed in 50 mM phosphate bufer (pH 6.0) containing 0.5% (*w/v*) of each substrate at 55 °C for 30 min. Values are the mean ± SD of three replicates.

*Af*Cel6A exhibited broad substrate specificity, including CM-Cellulose, Avicel^®^, xyloglucan, and birchwood xylan. This enzyme displayed higher specific activities toward CM-Cellulose (36.6 ± 2.1 U mg^−1^) and Avicel^®^ (35.8 ± 2.6 U mg^−1^) than birchwood xylan (21.1 ± 0.1 U mg^−1^) and xyloglucan (19.9 ± 0.3 U mg^−1^) (Figure 5). When CMC was the substrate, purified *Af*Cel6A had K_M_, V_max_, and *k*_cat_/K_M_ of 7.44 ± 0.51 g L^−1^, 195.2 ± 4.65 U mg^−1^, and 19.9 mL mg^−1^ s^−1^, respectively (Table 2).

We evaluated recombinant *Af*AA9_B peroxidase activity toward the chromogenic substrate 2,6-DMP and the co-substrate H_2_O_2_, according to Breslmayr et al. (2018) [80], with some modifications. Table 3 summarizes the kinetic parameters determined when the reactions were carried out at pH 6.0 or 9.0 and 50 °C.

The V_max_ values were higher at pH 9.0 for both the substrate (1481 ± 72.19 U g^−1^) and the co-substrate (972.5 ± 28.31 U g^−1^). Since we performed the saccharification tests at pH 6.0, we also determined the kinetic parameters under these conditions. At this pH, V_max_ was 78.52 ± 3.33 U g^−1^ for the substrate and 49.26 ± 4.48 U g^−1^ for the co-substrate. These results were expected because pH 9.0 was optimal for *Af*AA9_B activity.

Compared to the kinetic parameters described for other LPMOs, *Af*AA9_B had lower K_Mapp_ (0.79 µM) than PMO9D_SCYTH (0.51 mM), PMO9D_SCYTH (0.51 mM), and PMO9D_MALCI (1.17 mM), which showed that *Af*AA9_B had higher binding affinity for 2,6-DMP (Table 3).

### 2.5. Effect of Different Metal Ions and Chemicals

Cellobiohydrolases are commonly used in many industrial processes. The effects of additives and products of cellulose hydrolysis on the activity of these enzymes must be considered during operation on an industrial scale.

Table 4 depicts how different ions and reagents influence CM-Cellulose hydrolysis by purified *Af*Cel6A. At 5 mM, MnCl_2_ (189.25 ± 2.33%), DTT (150.68 ± 5.29%), CoCl_2_ (116.75 ± 1.36%), FeSO_4_ (125.83 ± 3.61%), β-mercaptoethanol (134.24 ± 1.02%), AgNO_3_ (179.27 ± 20.04%), and ascorbic acid (121.40 ± 2.55%) stimulated *Af*Cel6A activity. EDTA, DMSO, SLS, Triton X-100, Tween 20, CaCl_2_, MgSO_4_, KCl, and (NH_4_)_2_SO_4_ practically did not affect *Af*Cel6A activity. On the other hand, SDS inhibited the enzyme by approximately 50%. The fact that β-mercaptoethanol and DTT boosted *Af*Cel6A activity by 134.64% and 150.68%, respectively, suggested that the presence of sulfhydryl groups such as the ones from cysteine residues in the active site is important for enzymatic catalysis [81].

As for *Af*AA9_B, SLS (115.3 ± 0.7%), SDS (107.8 ± 4.8%), Tween 20 (103.7 ± 9.6%), DMSO (108.3 ± 1.5%), MgSO_4_ (113.9 ± 1.9%), and KCl (107.5 ± 2.1%) did not inhibit this enzyme. DTT, EDTA, CoCl_2_, FeSO_4_, and AgNO_3_ completely inhibited *Af*AA9_B. β-mercaptoethanol, ZnSO_4_, and CuSO_4_ decreased *Af*AA9_B activity by 70%. MnCl_2_, CaCl_2_, and (NH_4_)_2_SO_4_ affected *Af*AA9_B little.

We described that cellobiohydrolases act on short cellulose molecules and cellooligosaccharides, releasing disaccharide units, such as cellobiose [35]. Cellobiose is the major product of cellulose hydrolysis by cellobiohydrolases, whereas glucose is the final product of cellulose hydrolysis.

Product inhibition can affect lignocellulosic hydrolysis to glucose and represents a barrier to achieving the high product yields that are necessary for an efficient process [82].

**Figure 6:**
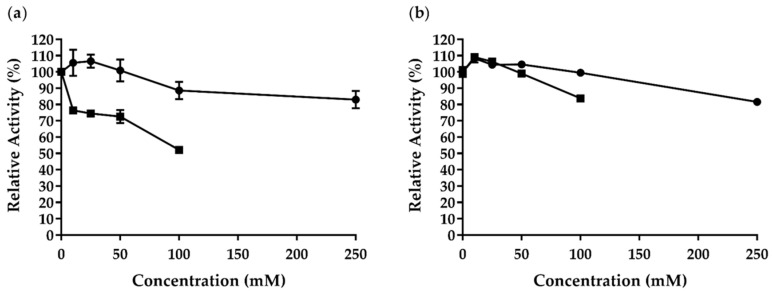
● Glucose and ■ cellobiose effects on (**a**) *Af*Cel6A and (**b**) *Af*AA9_B activity.

We examined how different glucose (10–250 mM) and cellobiose (10–100 mM) concentrations affected *Af*Cel6A activity (Figure 6a). Glucose at 100 and 250 mM inhibited the enzymatic activity by 12% and 13%, respectively. Cellobiose (100 mM) inhibited *Af*Cel6A activity by 50%. Cellobiohydrolase from *T. reesei* (*Cel6A*) has been described as the most efficient cellobiohydrolase, with IC_50_ of 240 mM for glucose and 20 mM for cellobiose [58]. Therefore, our results showed that *Af*Cel6A was more resistant to inhibition by both products because IC_50_ was higher than 250 mM for glucose and 100 mM for cellobiose.

Likewise, we investigated how both sugars affected *Af*AA9_B activity (Figure 6b). Surprisingly, the enzyme retained more than 80% of its initial activity when we added 250 mM glucose or 100 mM cellobiose to the reaction. Together, these findings indicated that *Af*Cel6A and *Af*AA9_B have potential application in enzymatic cellulose saccharification. However, to improve the efficiency of these enzymes and to increase glucose production, synergistic association with other enzymes is required.

### 2.6. Synergistic Action on Cellulose Hydrolysis

**Figure 7:**
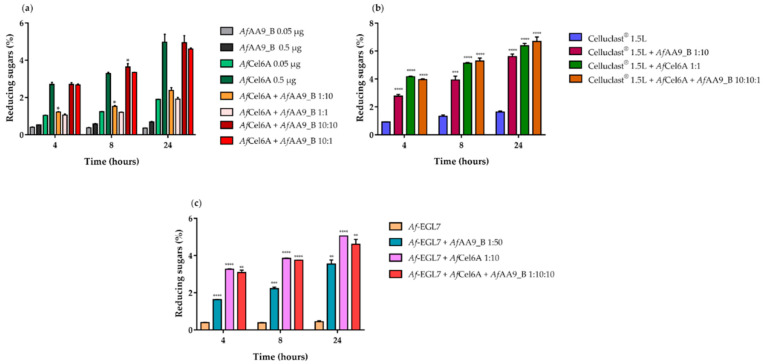
Synergistic action on 1% (*w/v*) CM-Cellulose of (**a**) *Af*Cel6A and *Af*AA9_B; (**b**) *Af*Cel6A, or *Af*AA9_B, or both with Celluclast^®^ 1.5 L cocktail; and (**c**) *Af*Cel6A, or *Af*AA9_B, or both with *Af*-EGL7. All reactions were incubated in 50 mM sodium phosphate buffer (pH 6.0) at 1000 rpm and 50 °C for 4, 8, or 24 h. At the end of each reaction, the measured reducing sugars were plotted as a function of the relative proportions among the added enzymes. Asterisks indicate significant difference (*p* < 0.05) in relation to the control system (*Af*Cel6A, *Af*-EGL7, or cocktail alone).

To determine the synergistic effects of *Af*Cel6A and *Af*AA9_B*,* we performed cellulose degradation experiments by using CMC as substrate. We conducted the reactions at different relative proportions and for different incubation times. Surprisingly, we observed no synergistic effect between *Af*Cel6A and *Af*AA9_B (Figure 7a).

We also investigated the synergistic effects between *Af*Cel6A and *Af*AA9_B and Celluclast^®^ 1.5L at different incubation times. Hydrolysis increased over time, and the yield of reducing sugars peaked after 24 h. Compared to the cocktail alone, *Af*AA9_B or *Af*Cel6A addition to the reaction mixture containing Celluclast^®^ 1.5L increased the release of reducing sugars by approximately 3.5 and 4.0 times, respectively. When Celluclast^®^ 1.5L cocktail was simultaneously associated with *Af*Cel6A and *Af*AA9_B at a ratio of 1:1:10, the maximum release of reducing sugars was 4.5 times higher compared to the cocktail alone. We verified a slight synergistic degree for Celluclast^®^ 1.5L cocktail, *Af*Cel6A, and *Af*AA9_B during CM-Cellulose hydrolysis. No inhibitory effect arose, probably because *Af*AA9_B acted synergistically with other enzymes in Celluclast^®^ 1.5L cocktail (Figure 7b).

LPMOs improve the efficiency of cellulase; i.e., endoglucanases and cellobiohydrolases, during cellulose hydrolysis, and they enhance cellulase adsorption and accessibility to cellulose [83,84]. We analyzed *Af*AA9_B and *Af*Cel6A synergism with endoglucanase *Af*-EGL7, which had been previously characterized [32]. Compared to *Af*-EGL7 alone, combination of *Af*-EGL7 and *Af*AA9_B released eight-fold more reducing sugars, whilst combination of *Af*-EGL7 and *Af*Cel6A increased hydrolyses by 11.5 times. When the three enzymes were associated at an *Af*-EGL7/*Af*AA9_B/*Af*Cel6A ratio of 1:10:10, 12.5 times more reducing sugars was released (Figure 7c). Thus, *Af*AA9_B acted synergistically with *Af*-EGL7, but not with *Af*Cel6A.

The efficiency of synergy among enzymes depends on the relative amount of crystalline to amorphous cellulose that is accessible within the substrate [85]. To evaluate how these enzymes acted on lignocellulosic biomass, we analyzed the associations of the enzymes in complex biomass, including SEB, rice straw, and corncob. SEB and corncob hydrolysis depended on time, but reducing sugars released from rice straw did not increase when we changed the reaction time from 24 to 48 h. Bernardi et al. (2019) [32] observed the same profile when they accomplished rice straw hydrolysis by a cocktail under similar conditions.

**Figure 8:**
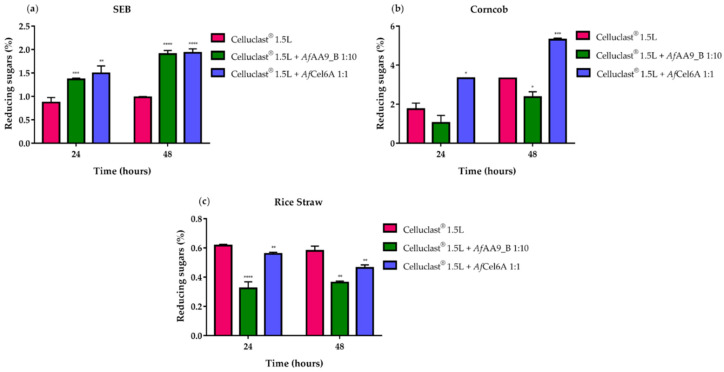
Effect of Celluclast^®^ 1.5L cocktail supplementation with *Af*Cel6A or *Af*AA9_B on hydrolysis of (**a**) SEB, (**b**) corncob, and (**c**) rice straw. All reactions were incubated in 50 mM sodium phosphate buffer (pH 6.0) containing 1% (*w/v*) of each biomass at 1000 rpm and 50 °C for 24 and 48 h. At the end of each reaction, the measured reducing sugars was plotted as a function of the relative proportions between the recombinant enzymes and commercial cellulases. Asterisks indicate significant difference (*p* < 0.05) in relation to the cocktail alone.

As shown in Figure 8a, compared to Celluclast^®^ 1.5L cocktail alone, *Af*Cel6A or *Af*AA9_B addition increased SEB hydrolysis by ~70% and ~95% after 24 and 48 h, respectively. Similarly, association between commercial cellulases and *Af*Cel6A boosted corncob hydrolysis by ~90% and ~70% after 24 and 48 h, respectively. On the other hand, *Af*AA9_B addition seemed to affect hydrolysis negatively (Figure 8b). The same inhibitory effect of LPMOs has been observed on rice straw, while *Af*Cel6A addition almost did not impact the release of reducing sugars (Figure 8c). The divergent results among the three agricultural residues pointed to the substrate-dependence and substrate specificity of *Af*Cel6A and *Af*AA9_B synergism with cellulases [86].

**Figure 9:**
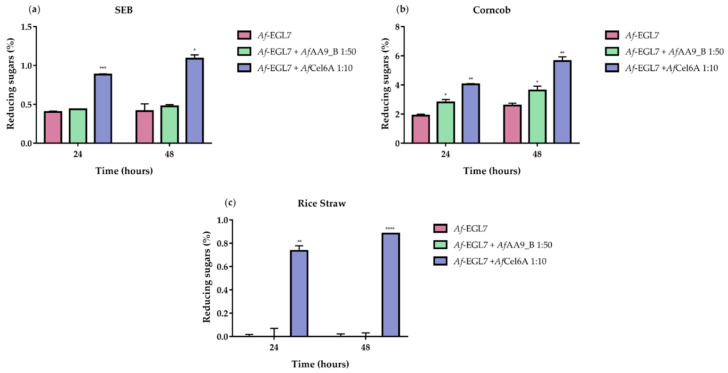
Combined activities of *Af*-EGL7 and *Af*Cel6A or *Af*AA9_B on the hydrolysis of (**a**) SEB; (**b**) corncob; and (**c**) rice straw. All reactions were incubated in 50 mM sodium phosphate buffer (pH 6.0) containing 1% (*w/v*) of each biomass for 24 and 48 h at 50 °C and 1000 rpm. At the end of each reaction, the measured reducing sugars were plotted in function of the relative proportions between the recombinant enzymes. Asterisks indicate significant difference (*p* < 0.05) in relation to the *Af*-EGL7 alone.

Compared to *Af*-EGL7 alone, the association between *Af*-EGL7 and *Af*Cel6A increased the amount of reducing sugars released from the three biomasses: ~163%, ~118%, and ~88% for SEB (Figure 9a), corncob (Figure 9b), and rice straw (Figure 9c), respectively, after 48 h. The *Af*-EGL7 *Af*AA9_B combination also improved SEB and corncob hydrolysis, but it did not affect rice straw degradation.

## 3. Materials and Methods

### 3.1. Strains, Culture Conditions, and Vectors

Mycelia of *Aspergillus fumigatus* Af293 (kindly donated by Professor Sérgio Akira Uyemura—University of São Paulo, Ribeirão Preto, Brazil) were obtained for RNA extraction. Fresh conidia (2 × 10^6^ per mL) were inoculated in YNB minimal medium (1× salt solution, 0.1% (*v*/*v*) trace elements, and 0.05% (*w/v*) yeast extract) containing 1% (*w/v*) fructose and incubated under shaking at 200 rpm and 37 °C for 16 h. The mycelia were harvested, washed, and transferred to YNB medium containing 1% (*w/v*) sugarcane exploded bagasse (SEB) at 200 rpm and 37 °C for 24 h.

*E. coli* DH10β was used to clone and to propagate the recombinant vectors. The strain was kept in Luria–Bertani medium supplemented with the appropriate antibiotic.

*Pichia pastoris* strain X-33 (Invitrogen, Carlsbad, CA, USA) was used to produce the heterologous proteins. The employed growth conditions are described in the EasySelect™ Pichia Expression Kit manual (Invitrogen, Carlsbad, CA, USA).

The plasmids pPICZB and pPICZαA (Invitrogen, Carlsbad, CA, USA) were used to clone, to sequence, and to express *Af*AA9_B and *Af*Cel6A, respectively.

Xyloglucan from tamarind seed and xylan from beechwood were acquired from Megazyme (Megazyme International, Bray, Co., Wicklow, Ireland). Avicel^®^ PH-101 and low-viscosity CM-Cellulose (CMC) were purchased from Sigma (Sigma–Aldrich, St. Louis, MO, USA).

Biomasses (rice straw and corncob) were provided by Professor Maria de Lourdes Teixeira de Moraes Polizeli (University of São Paulo, Ribeirão Preto, Brazil). Sugarcane exploded bagasse (SEB) was provided by Professor João Atílio Jorge (University of São Paulo, Ribeirão Preto, Brazil).

### 3.2. RNA Extraction, cDNA Synthesis, and Gene Amplification

Total RNA from *A. fumigatus* mycelia was isolated by using the Direct-zol™ RNA MiniPrep kit (Zymo Research, Irvine, CA, USA); the manufacturer’s instructions were followed. cDNA was synthesized by using SuperScript^®^ II Reverse Transcriptase (Invitrogen, Carlsbad, CA, USA).

Table 5 describes the specific primer sequences obtained for *AfAA9_B* and *AfCel6A* amplification and cloning into the vectors pPICZB and pPICZαA, respectively:

The amplification reactions were performed with Phusion High-Fidelity DNA Polymerase (Thermo Fisher Scientific, Waltham, MS, USA), and the PCR product was analyzed by electrophoresis and purified from 1% (*w/v*) agarose gel by using the QIAquick Gel Extraction kit (Qiagen, Hilden, Germany).

### 3.3. Enzyme Production and Purification

*AfAA9_B* and *AfCel6A* ORFs (open reading frames) with and without predicted signal peptides, respectively, were cloned into the corresponding vectors pPICZB and pPICZαA (previously digested with the restriction enzymes *Eco*RI and *Xba*I) by the circular polymerase extension cloning (CPEC) method [87]. Both CPEC reactions were carried out with Phusion High-Fidelity DNA Polymerase (Thermo Scientific). The thermocycling conditions were as follows: 98 °C for 30 s; 35 cycles of 98 °C for 10 s, 55 °C for 30 s, and 72 °C for 2 min 30 s; and 72 °C for 10 min. The cloning products were transformed to *E. coli* DH10β, and the resistant transformants were selected with zeocin (50 µg mL^−1^). Next, the recombinant vectors pPICZB/*AfAA9_B* and pPICZαA/*AfCel6A* were linearized with *Pme*I and transformed into competent *P. pastoris* X-33 cells by electroporation according to the EasySelect™ Pichia Expression Kit manual (Invitrogen).

Zeocin-resistant *P. pastoris* transformants were selected to produce the enzymes. The recombinant yeasts were cultivated in buffered glycerol-complex medium (BMGY) at 240 rpm and 30 °C. For heterologous *Af*AA9_B expression, *P. pastoris* cells were resuspended in buffered methanol-complex medium (BMMY). Methanol (1% (*v*/*v*)) was added to the medium at 24-h intervals for six days, and the supernatant was harvested from the grown culture. The supernatant containing secreted recombinant enzyme (*Af*AA9_B) was concentrated 10 times by using an Amicon Ultra-15 Centrifugal Filter—10-kDa cutoff (Millipore, Burlington, MS, USA). Protein expression was verified by SDS-PAGE.

*Af*Cel6A was expressed as described above, but 1.5% (*v*/*v*) methanol was added.

To purify the enzymes, the concentrates were resuspended in 20 mM sodium phosphate buffer containing 500 mM NaCl (pH 7.4) and loaded onto Ni^+^ Sepharose 6 Fast Flow resin (Ge Healthcare, Little Chalfont, UK). An imidazole gradient from 0 to 500 mM was applied to the columns to elute the recombinants His6-tagged *Af*AA9_B and His6-tagged *Af*Cel6A. The fractions were collected, and the enzymes were analyzed by 10% (*w/v*) SDS-PAGE, stained with Comassie Brilliant Blue R-250 (Sigma–Aldrich, St. Louis, MO, USA). Fractions containing the recombinant enzymes were mixed and buffer-exchanged by using an Amicon Ultra-15 Centrifugal Filter—10 kDa cutoff (Millipore) to remove excess imidazole.

To coordinate copper to the *Af*AA9_B active site, the purified recombinant enzyme was incubated with CuSO_4_ at 1:3 molar ratio and 4 °C for 30 min. Then, the *Af*AA9_B solution was dialyzed against 20 mM sodium phosphate buffer containing 500 mM NaCl (pH 7.4) under shaking at 4 °C for 48 h, to remove traces of non-coordinated Cu^2+^. The purified *Af*AA9_B concentration was determined by the Greenberg method [88].

The *Af*AA9_B band from the SDS-PAGE gel was manually excised, reduced, alkylated, digested with trypsin, purified (Promega, Madison, WI, EUA—V5111), and analyzed by mass spectrometry according to a previously described method [89].

### 3.4. Glycosylation

N-glycosylation sites were predicted by employing NetNGlyc 1.0 (http://www.cbs.dtu.dk/services/NetNGlyc/), and O-glycosylation was analyzed by using NetOGlyc 4.0 (http://www.cbs.dtu.dk/services/NetOGlyc/). Deglycosylation of recombinant *Af*AA9_B and *Af*Cel6A was accomplished by using Endoglycosidase H (Endo H, New England Biolabs, Ipswich, MA, USA) in non-denaturing conditions, as per the manufacturer’s procedure. The resulting enzymes were further analyzed by SDS-PAGE.

### 3.5. Structural Analysis by Circular Dichroism (CD)

Circular dichroism (CD) spectra of the enzymes were obtained between 190 and 250 nm (far-UV) on a JASCO-810 spectropolarimeter; quartz cuvettes with optical path of 0.1 cm were employed. *Af*AA9_B (0.021 mg mL^−1^) and *Af*Cel6A (0.0026 mg mL^−1^) were diluted in 20 mM sodium phosphate (pH 7.4), and the readings were performed in quadruplicate at scanning speed, band width, and D.I.T. of 50 nm min^−1^, 3 nm, and 1 s, respectively. All the spectra were corrected for the buffer contributions and converted from millidegrees (mdeg) to Δε in M^−1^ cm^−1^ according to the following equation: Δε = θ [(0.1⋅MRW)/(d⋅c⋅3298)], where θ is the ellipticity value originally given by equipment (millidegrees), MRW is the enzyme mean residual weight, d is the optical path (cm), and c is the enzyme concentration (mg mL^−1^). All the secondary structures of the enzymes were predicted by using the BeStSel web server [53], and the results were compared with structures modeled on the Phyre2 [55] and Discovery Studio [90] web servers.

### 3.6. LPMO Activity Assay

Purified *Af*AA9_B activity was analyzed as reported by Breslmayr et al. (2018) [80]. The assay consisted of a reaction mixture containing 1 mM 2,6-dimethoxyphenol (2,6-DMP) (Sigma–Aldrich, St. Louis, MO, USA), 100 μM H_2_O_2_, and recombinant purified *Af*AA9_B in 50 mM sodium phosphate buffer (pH 8.0). For the blank, the enzyme was denatured by incubation at 99 °C for 30 min before the reaction mixture was added. After 5 min at 30 °C, absorbance was read at 469 nm to calculate the LPMO peroxidase activity.

### 3.7. AfCel6A Activity Assay

*Af*Cel6A activity was determined by measuring reducing sugars from the reaction by the 3,5-dinitrosalicylic acid (DNS) method [91]. Briefly, the reaction mixture consisting of 1% CM-Cellulose (*w/v*) in 50 mM sodium phosphate buffer (pH 6.0) was incubated at 55 °C for 30–45 min. The enzymatic action was stopped by adding an equal volume of the DNS reagent. The mixture was boiled for 5 min and cooled, and absorbance was measured at 540 nm. One unit of *Af*Cel6A was defined as the amount of enzyme that released 1 µmol of reducing sugar from the substrate per minute. Each assay was carried out in triplicate. Enzyme concentration was determined by the Greenberg method [88].

### 3.8. Enzymatic Properties of AfAA9_B and AfCel6A

The optimal pH for *Af*AA9_B activity was measured at pH ranging from 4.0 to 8.0 in McIlvaine buffer (citric acid-Na_2_HPO_4_) and at pH 9.0 and 10.0 in 100 mM Glycine-NaOH buffer at 30 °C. The relative activity was calculated with respect to the maximum activity of 100%; the aforementioned method was followed. The pH stability was estimated by measuring the residual enzymatic activity after the enzyme was incubated without substrate in the aforementioned buffers at pH ranging from 3.0 to 10.0 and 4 °C for up to 72 h. To determine the *Af*AA9_B thermal stability, the enzyme was preincubated without substrate at 50 and 60 °C for up to 72 h. To measure the residual activity, the enzymatic activity without preincubation was considered 100%.

The optimal pH for *Af*Cel6A activity was measured from 3.0 to 8.0 in McIlvaine buffer (citric acid-Na_2_HPO_4_) at 55 °C. The optimal temperature was examined between 40 and 80 °C. The relative activity was calculated with respect to the maximum exhibited activity of 100%; the aforementioned method was followed.

The *Af*Cel6A pH stability was estimated by measuring the residual enzymatic activity under standard conditions after the enzyme was incubated without substrate in Mcllvaine (citrate–phosphate) buffers pH 3.0–8.0 and in 100 mM Glycine-NaOH buffers pH 9.0 and 10.0 at 4 °C for up to 72 h. To determine the *Af*Cel6A thermal stability, the enzyme was preincubated without substrate at temperatures ranging from 50 to 90 °C for different times. To measure the residual activity, the enzymatic activity without preincubation was considered 100%.

### 3.9. Effect of Additives

How various metal ions affected *Af*AA9_B and *Af*Cel6A was determined by adding Mn^2+^, Co^2+^, Ca^2+^, Fe^2+^, Zn^2+^, Mg^2+^, Cu^2+^, NH_4_^+^, K^+^, or Ag^+^ at a final concentration of 5 mM to the reaction mixture. The effects of EDTA, SDS, Tween 20, Triton X-100, SLS, β-mercaptoethanol, DTT, and DMSO were also tested. For *Af*Cel6A, the effect of ascorbic acid addition was also evaluated. Control reactions (100% activity) were performed without any additive. The relative activity was estimated as compared to the controls.

### 3.10. Glucose and Cellobiose Effects on AfCel6A and AfAA9_B Activity

The glucose (10–250 mM) and cellobiose (up to 100 mM) effects on the activity of *Af*AA9_B and *Af*Cel6A were determined in the presence of increasing concentrations of both sugars by using the chromogenic substrates 2,6-DMP and 4-nitrophenyl β-D-cellobioside (Sigma–Aldrich, St. Louis, MO, USA), respectively.

### 3.11. Kinetic Assays

The *Af*AA9_B kinetic parameters (K_M_, V_max_, and *k_cat_*) were determined for the substrate 2,6-DMP (0.1 to 10 mM) and the co-substrate H_2_O_2_ (1 to 500 µM). The reactions were performed in 50 mM sodium phosphate buffer (pH 6.0) and 100 mM glycine-NaOH buffer (pH 9.0) at 50 °C. The parameters were calculated by Michaelis–Menten nonlinear regression.

The *Af*Cel6A kinetic parameters were determined when CM-Cellulose (0.5–30 mg mL^−1^) was used as substrate. The reactions were performed in 50 mM sodium phosphate buffer (pH 6.0) as previously described. The parameters were calculated by the Michaelis-Menten nonlinear regression graphical method.

### 3.12. Combined Assays

*Af*AA9_B and *Af*Cel6A enzymatic assays were carried out concomitantly with the recombinant endoglucanase *Af*-EGL7 as previously described [32]. The assays were performed by adding 1 µg of *Af*-EGL7 to 50 µg of *Af*AA9_B (1:50) or 10 µg of *Af*Cel6A (1:10) per gram of substrate. The reaction mixtures consisted of CM-Cellulose (1% (*w/v*)) in 50 mM sodium phosphate buffer (pH 6.0) containing 1 mM ascorbic acid in a final volume of 1 mL. The reactions were performed in a thermomixer (Eppendorf) at 50 °C and 1000 rpm for 4, 8, or 24 h.

In the same way, *Af*AA9_B and *Af*Cel6A were combined at different concentration proportions (10:1, 1:1, 10:10, or 1:10), where the minimum and maximum enzyme loading corresponded to 5 and 50 µg of added enzyme per gram of CM-Cellulose, respectively.

Finally, the effect of the simultaneous association of the three recombinant enzymes on the degradation of CM-Cellulose was evaluated. While the *Af*-EGL7 concentration was 1 µg g^−1^, the concentrations of both *Af*AA9_B and *Af*Cel6A were 10 µg per gram of CM-Cellulose, generating the ratio 1:10:10. The reactions were carried out as described above.

The degradation efficiencies were assessed by estimating the released reducing sugars by the DNS method. The reported results represent the mean ± SD calculated from at least three experimental replicates.

### 3.13. Synergistic Activity with Celluclast^®^ 1.5L

*Af*AA9_B and *Af*Cel6A synergistic activity of during enzymatic hydrolysis was investigated in combination with Celluclast^®^ 1.5L, a commercial cellulase cocktail from *Trichoderma reesei*.

To this end, 0.05 FPU of Celluclast^®^ 1.5L cocktail was associated with 50 µg of *Af*AA9_B (ratio 1:10) or 5 µg of *Af*Cel6A (ratio 1:1) per gram of CM-Cellulose (1% (*w/v*) in 50 mM sodium phosphate buffer (pH 6.0) containing 1 mM ascorbic acid. The reactions were conducted at 1000 rpm and 50 °C for up to 24 h in a final volume of 1 mL.

The effect of the simultaneous association between commercial cellulases and the two recombinant enzymes from *A. fumigatus* on the degradation of CM-Cellulose was also evaluated. While the Celluclast^®^ 1.5 L cocktail loading was fixed at 0.05 FPU g^−1^, the *Af*Cel6A and *Af*AA9_B concentrations were 5 and 0.5 µg of enzyme added per gram of CM-Cellulose, respectively. The reactions were carried out as described above.

The percent hydrolysis yields were determined by estimating the released reducing sugars by the DNS method [91]. The reported results represent the mean ± SD calculated from at least three experimental replicates.

### 3.14. Lignocellulosic Biomass Saccharification

Enzymatic hydrolyses of some agro-industrial residues were carried out as described by Bernardi et al. (2019) with some modifications [32]. Saccharification was accomplished in 50 mM sodium phosphate buffer (pH 6.0) containing 1% (*w/v*) of one of the following biomasses: SEB (sugarcane exploded bagasse), rice straw, or corncob.

Different associations between the enzymes were used during biomass saccharifications. *Af*-EGL7 (18 µg g^−1^) was combined with *Af*AA9_B (900 µg g^−1^) or *Af*Cel6A (180 µg g^−1^). Similarly, a fixed concentration of Celluclast 1.5L cocktail (0.9 FPU g^−1^) was associated with *Af*AA9_B (900 µg g^−1^) or *Af*Cel6A (90 µg g^−1^). The reactions were conducted at 1000 rpm and 50 °C for up to 48 h in a final volume of 1 mL. DNS was added to stop the reactions and to measure the released reducing sugars. The reported results represent the mean ± SD calculated from at least three experimental replicates.

### 3.15. Reproducibility of the Results

All the data are the mean of at least three independent experiments and show consistent results.

## 4. Conclusions

Novel cellobiohydrolase and LPMO from *Aspergillus fumigatus* were characterized after they were expressed in *P. pastoris*. Supplementation of a cellulase cocktail with both enzymes improved the yield of saccharification of different biomasses, especially SEB. However, *Af*AA9_B did not have a positive effect on *Af*Cel6A activity. On the other hand, *Af*AA9_B acted synergistically with endoglucanase *Af*-EGL7. These different synergistic effects are important to understand the action of LPMOs with cellulases and would help to design new commercial enzymatic cocktails. Considering the reduction of costs in lignocellulose conversion, we can conclude that supplementation of Celluclast^®^ 1.5L with *Af*Cel6A or *Af*AA9_B suffices to increase the hydrolytic activity, so the composition of cellulase cocktails may need to be reconsidered.

## Figures and Tables

**Table 1:** Peptide sequences.

Protein Gene	Peptide Sequence	Precursor M_Z_	Precursor Charge	Product M_Z_	Product Charge
AFUA_4G07850	ITSIAGLLASASLVAGHGFVSGIVADGK	871.675226	3	1049.562586	1
AFUA_4G07850	ITSIAGLLASASLVAGHGFVSGIVADGK	871.675226	3	992.541122	1
AFUA_4G07850	ITSIAGLLASASLVAGHGFVSGIVADGK	871.675226	3	845.472708	1
AFUA_4G07850	ITSIAGLLASASLVAGHGFVSGIVADGK	871.675226	3	746.404294	1
AFUA_4G07850	ITSIAGLLASASLVAGHGFVSGIVADGK	871.675226	3	659.372266	1
AFUA_4G07850	ITSIAGLLASASLVAGHGFVSGIVADGK	871.675226	3	602.350802	1
AFUA_4G07850	NTDPGIK	372.912411	2	630.345717	1
AFUA_4G07850	NTDPGIK	372.912411	2	529.298038	1
AFUA_4G07850	NTDPGIK	372.912411	2	414.271095	1
AFUA_4G07850	NTDPGIK	372.912411	2	317.218332	1

**Table 2:** Comparison among catalytic and biochemical properties of GH6 cellobiohydrolases.

Source Organism	Enzyme Name	Expression System	Substrate	V_max_	K_M_	*k_cat_*	*k_cat_*/K_M_	Optimal T	Optimal pH	Thermal Stability	pH Stability	Ref.
*Aspergillus fumigatus* Af293	*Af*Cel6A	*Pichia pastoris*	CMC-Na	195.2 ± 4.65 U mg^−1^	7.44 ± 0.51 g/L	147.9 s^−1^	19.9 mL mg^−1^ s^−1^	55–60 °C	pH 5.5–6.5	>70% after 24 h at 50 °C; about 40% after 90 min at 60–80 °C; more than 25% after 30 min at 90 °C	More than 70% at pH 3.0–10.0 after 72 h	This study
*Aspergillus fumigatus*	*Af*Cel6A	*Aspergillus oryzae*	Avicel PH101	-	48.6 ± 14.8 g L^−1^	0.9 ± 0.1 s^−1^	-	70 °C	-	No loss at 60 °C after 1 h	-	[35]
*Aspergillus terreus*	*At*Cel6A	*Aspergillus oryzae*	Avicel PH101	-	-	-	-	50 °C	-	>90% after 1 h at 50 °C	-	[35]
*Talaromyces funiculosus*	*Tf*Cel6A	*Aspergillus oryzae*	Avicel PH101	-	21.6 ± 3.2 g L^−1^	0.5 ± 0.02 s^−1^	-	60 °C	-	No loss at 50 °C after 1 h	-	[35]
*Colletotrichum graminicola*	*Cg*Cel6A	*Aspergillus oryzae*	Avicel PH101	-	-	-	-	40 ° C	-	>90% after 1 h at 40 °C	-	[35]
	*Cg*Cel6B				89.0 ± 13.2 g L^−1^	1.8 ± 0.2 s^−1^		50 °C		>90% after 1 h at 50 °C		
*Trichoderma reesei*	*Tr*Cel6A	*Aspergillus oryzae*	Avicel PH101	-	24.3 ± 4.0 g L^−1^	0.6 ± 0.04 s^−1^	-	70 °C	-	No loss at 50 °C after 1 h	-	[35]
	Cel6A^1^	*Pichia pastoris*	CMC-Na	10.7 mmol min^−1^ mg^−1^	0.31 mg mL^−1^	-	-	60 °C	pH 5.5	90% after 30 min at 60 °C	-	[59]
	Cel6A^2^	*Pichia pastoris*	PASC	-	-	-	-	55 °C	pH 5.5–6.0	100% at 40 °C and 50% at 60 °C, after 30 min	No loss at pH 5.0–6.0; rapid inactivation at more alkaline pH; and some instability at more acidic pH after 30 min	[60]
	CBHII	-	PASC	10 U mg^−1^	3.8 mg mL^−1^	-	-	60 °C	pH 5.0	80% after 30 min at 60 °C	Stable at pH 3.5–6.0 after 30 min	[61]
*Magnaporthe oryzae* Ina72	*Mo*Cel6A	*Magnaporthe oryzae*	Cellotetraose	454.5 µg min^−1^ mg^−1^	24.3 mM	-	-	40 °C	pH 9.0	-	-	[62]
			Cellopentaose	63.3 µg min^−1^ mg^−1^	3.3 mM							
*Schizophyllum commune* KMJ820	CBH II	*Escherichia coli*	pNPC	20.8 U mg^−1^	1.4 mM	-	-	50 °C	pH 5.0	-	-	[63]
*Penicillium occitanis* Pol 6	CBH II	-	pNPC	-	5 mM	-	-	65 °C	pH 4.0–5.0	Almost 100% at 30–50 °C; 50% at 60 °C; and complete inactivation at 70 °C, after 30 min	Stable at pH 2.0–9.0 after 24 h	[64]
*Talaromyces emersonii*	CBH II	-	CNPG_3_	9.1 U mg^−1^	4.5 mM	8.9 s^−1^	1.9 mM^−1^ s^−1^	68 °C	pH 3.8	t_1/2_ = 38 min at 80 °C (pH 5.0)	t_1/2_ = 38 min at pH 5.0 (80 °C)	[65]
*Trichoderma viride* CICC13038	CBH II	*Saccharomyces cerevisiae*	CMC-Na	-	-	-	-	70 °C	pH 5.0	-	-	[66]
*Neocallimastix patriciarum* J11	J11 CelA	*Escherichia coli*	Barley β-glucan	-	-	-	-	50 °C	pH 6.0	More than 70% at up to 50 °C and approximately 50% at 70 °C, after 1 h	More than 80% at pH 5.2–11.3; and approximately 70% at pH 3.0, 4.2, and 12.3, after 1 h	[67]
*Irpex lacteus* MC-2	Ex-4	*Pichia pastoris*	PASC	-	-	-	-	50 °C	pH 5.0	More than 80% at 60 °C (pH 3.0–8.0) after 1 h	More than 80% at pH 3.0–8.0 (60 °C) after 1 h	[68]
*Chaetomium thermophilum* HSAUP_07_2651	CBH II	*Pichia pastoris*	pNPC	-	-	-	-	50 °C	pH 4.0	No loss at 50 °C; approximately 20% at 60 °C; and complete inactivation at 80 °C, after 1 h	-	[69]

**Table 3:** Comparison among catalytic and biochemical properties of LPMOs.

Source Organism	Protein Name	Expression System	Substrate	Co-Substrate	V_max_	K_M_	*k_cat_*	*k_cat_*/K_M_	Optimal T	Optimal pH	Thermostability	pH Stability	Ref
*Aspergillus fumigatus* Af293	*Af*AA9_B	*Pichia pastoris* X33	2,6-DMP	§ ^a^ H_2_O_2_	78.52 ± 3.33 U g^−1^	2.04 ± 0.24 µM	0.034 s^−1^	0.017 µM^−1^ s^−1^	-	*** 9	-	-	This study
				§ ^b^ H_2_O_2_	1481 ± 72.19 U g^−1^	0.79 ± 0.12 µM	0.64 s^−1^	0.81 µM^−1^ s^−1^	-		60 °C: 50 % after 48 h 50 °C: almost 100% of activity after 48 h	No loss of activity at pH 5.0–10.0	
			§ 2,6-DMP	^a^ H_2_O_2_	49.26 ± 4.48 U g^−1^	106.3 ± 27.9 µM	0.021 s^−1^	1,98 × 10^−4^ µM^−1^ s^−1^	-	-	-	-	
				^b^ H_2_O_2_	972.5 ± 28.31 U g^−1^	12.15 ± 1.76 µM	0.42 s^−1^	0.035 µM^−1^ s^−1^	-	-	-	-	
*Scytalidium thermophilum*	PMO9D_SCYTH	*Pichia pastoris* X33	Avicel	§ H_2_O_2_	0.36 U mg^−1^	4.54 mg mL^−1^	2.99 × 10^−2^ min^−1^	6.58 × 10^−3^ mg^−1^ mL min^−1^	-	-	-	-	[70]
			CMC		14.96 U mg^−1^	10.6 mg mL^−1^	1.61 min^−1^	1.52 × 10^−1^ mg^−1^ mL min^−1^	60 °C	7	60 °C (t_1/2_ = 60.58 h, pH 7.0)	Above 90% after 48 h at pH 7.0	
			2,6-DMP		0.13 U mg^−1^	0.51 mM	1.84 × 10^−1^ min^−1^	3.57 × 10^−1^ mM^−1^ min^−1^		-	-	-	
*Malbranchea cinnamomea*	PMO9D_MALCI		Avicel		0.17 U mg^−1^	5.87 mg mL^−1^	1.05 × 10^−2^ min^−1^	1.79 × 10^−3^ mg^−1^ mL min^−1^		-	-	-	
			CMC		9.59 U mg^−1^	29.27 mg mL^−1^	0.76 min^−1^	2.62 × 10^−2^ mg^−1^ mL min^−1^	50 °C	9	50 °C (t_1/2_ = 144 h, pH 7.0)	Above 80% after 72 h at pH 9.0	
			2,6-DMP		0.12 U mg^−1^	1.17 mM	1.21 min^−1^	1.03 × 10^−1^ mM^−1^ min^−1^		-	-	-	
*Thielavia terrestris*	*Tt*LPMO9E	-	PWS	§ O_2_	-	49.80 g L^−1^	3.8 min^−1^	* 1.85 × 10^3^ M^−1^ min^−1^	-	-	-	-	[71]
*Myceliophthora thermophila*	*Mt*PMO9E	*Neurospora crassa*	cellohexaose	§ O_2_	-	32 µM	10.1 min^−1^	0.30 µM^−1^ min^−1^	-	-	-	-	[72,73]
			§ cellohexaose	O_2_	-	230 µM	17 min^−1^	7.4 × 10^−2^ µM^−1^ min^−1^	-	-	-	-	
				H_2_O_2_	-	53 µM	# 15.9 s^−1^	3.0 × 10^5^ M^−1^ s^−1^	-	-	-	-	
*Serratia marcescens*	CBP21	*Escherichia coli* BL21(DE3) Star	CNW	§ H_2_O_2_	1.11 µM s^−1^	0.58 mg mL^−1^	6.7 s^−1^	≅10^6^ M^−1^ s^−1^	-	-	-	-	[74]
			§ CNW	H_2_O_2_	-	2.8 µM	-	-	-	-	-	-	
*Lentinus similis*	*Ls*(AA9)A	*Aspergillus oryzae* MT3568	cellotetraose	§ O_2_	-	43 µM	0.11 s^−1^	2.6 × 10^3^ M^−1^ s^−1^	-	-	-	-	[75]
*Aspergillus fumigatus* NITDGPKA3	** CAF32158.1	*Pichia pastoris* X33	2,6-DMP	§ H_2_O_2_	1.11 µM min^−1^	11.23 µM	0.642 min^−1^	5.7 × 10^−2^ µM min^−1^	-	-	-	-	[76]
*Myceliophthora thermophila*	*Mt*LPMO9D	*Myceliophthora thermophila* C1	-	-	-	-	-	-	-	-	**** Tm_app_ at pH 7.0 = 68 °C	-	[77]
*Myceliophthora thermophila*	*Mt*LPMO9J	*Aspergillus nidulans*	-	-	-	-	-	-	-	-	**** Tm_app_ at pH 6.0 = 58 °C	-	[78]
*Thermoascus aurantiacus*	*Ta*LPMO9A	*Aspergillus oryzae*	-	-	-	-	-	-	-	-	**** Tm_app_ at pH 7.0 = 69 °C	-	[79]

Notations: (§) fixed concentration; (*) *k_cat_*/K_M_ calculated for this paper considering the molecular weight of 24.2 kDa for *Tt*LPMO9E; (**) this enzyme has no name yet, the provided code is its codifying gene; (***) pH stability not correlated with analyzed pH values; (****) apparent midpoint transition temperatures (Tm_app_) calculated on the basis of CD (*Mt*LPMO9D and *Ta*LPMO9A) and Intrinsic Trp fluorescence emission (ITFE) (*Mt*LPMO9J) analysis; (#) *k_cat_* estimated based on previous *k_cat_*/K_M_ and K_M_ values for *Mt*PMO9E. Kinetic studies were conducted at (^a^) pH = 6.0 and (^b^) pH = 9.0. Abbreviations: PWS—Pretreated wheat straw; CNW—Chitin nanowhisker. For more details, see the corresponding references.

**Table 4:** Effects of additives on *Af*Cel6A and *Af*AA9_B activity.

	*Af*AA9_B	*AfCel6A*
Additive	% Relative Activity	
None	100.0 ± 1.0	100.0 ± 0.9
SDS	107.8 ± 4.8	53.72 ± 0.31
Tween 20	103.7 ± 9.6	93.24 ± 2.11
EDTA	0	91.88 ± 1.56
Ascorbic Acid	-	121.40 ± 2.55
DMSO	108.3 ± 1.5	101.03 ± 3.58
β-mercaptoethanol	36.7 ± 0.6	134.24 ± 1.02
ZnSO_4_	36.8 ± 6.1	83.67 ± 0.65
MnCl_2_	82.2 ± 0.7	189.25 ± 2.33
CoCl_2_	0	116.75 ± 1.36
CaCl_2_	93.6 ± 3.3	101.1 ± 2.4
FeSO_4_	0	125.83 ± 3.61
MgSO_4_	113.9 ± 1.9	95.92 ± 2.13
CuSO_4_	35.2 ± 4.6	85.08 ± 0.65
AgNO_3_	0	179.27 ± 20.04
KCl	107.5 ± 2.1	98.87 ± 2.44
(NH_4_)_2_SO_4_	88.9 ± 3.2	99.9 ± 2.1
DTT	0	150.68 ± 5.29
Triton X-100	84.8 ± 2.3	91.55 ± 1.19
SLS	115.3 ± 0.7	93.80 ± 1.60

**Table 5:** Primer sequences used to amplify and to clone genes.

Primer Name	Sequence (5′-3′)
*Af**AA9_B*** Fw	**CAAAAAACAACTAATTATTCGAAACGAGGAATTCC**ATGACTTTGTCCAAGATCAC
*Af**AA9_B*** Rv	**CAGATCCTCTTCTGAGATGAGTTTTTGTTCTAG**AGCGTTGAACAGTGCAGGAC
*Af**Cel6A*** Fw	**GAGAAAAGAGAGGCTGAAGCTGAATTC**CAGCAGACCGTATGG
*Af**Cel6A*** Rv	**ATCCTCTTCTGAGATGAGTTTTTGTTCTAG**AAAGGACGGGTTAGC

Notation: The overlapping regions between the vector and the insert are in bold.

## Data Availability

Data sharing is not applicable to this article.

## Supplementary Materials

Supplementary Materials can be found at https://www.mdpi.com/1422-0067/22/1/276/s1, Table S1: Secondary structure proportions of AfAA9_B and AfCel6A from literature and different prediction methods in comparison with the one determined by BeStSel based on the CD spectra.

## Abbreviations

2G ethanol	Second-generation ethanol
LPMO	Lytic Polysaccharide Monooxygenase
AA	Auxiliary Activity
GH	Glycoside Hydrolase
CD	Circular Dichroism
CBM	Carbohydrate Binding Module
CMC-Na	Sodium Carboxymethyl Cellulose
2,6-DMP	2,6-Dimethoxyphenol
PASC	Phosphoric-acid Swollen Cellulose
pNPC	4(*p*)-nitrophenyl β-D-cellobioside
SEB	Sugarcane Exploded Bagasse
DNS	3,5-Dinitrosalicylic acid
FPU	Filter Paper Unit
