# Supplementary material for: LPMO AfAA9_B and Cellobiohydrolase AfCel6A from A. fumigatus Boost Enzymatic Saccharification Activity of Cellulase Cocktail
Source: Int J Mol Sci. 2020 Dec 29;22(1):276. doi: 10.3390/ijms22010276 (PMC7795096; doi:10.3390/ijms22010276)
Supplement: Supplementary file 1 [file ijms-22-00276-s001.pdf]

Supplementary Table S1: Secondary structure proportions of *Af*AA9_B and *Af*Cel6A from literature and different prediction methods in comparison with the one determined by BeStSel based on the CD spectra.

	***Af*AA9_B**				***Af*Cel6A**		
	BeStSel (CD)	PDB: 5X6A	Phyre2	Kabsch & Sander	BeStSel (CD)	Phyre2	Kabsch & Sander
**α-helix (%)**	8.3	12.7	1.0	10.9	27.0	28.0	30.3
**β-strand (%)**	31.4	31.4	40.0	29.3	7.7	10.0	10.8
**Turns (%)**	11.9	-	-	-	12.8	-	-
**Others (%)**	48.4	55.9	59.0	59.8	52.8	62.0	58.9
